# Shoot Characterization of Isoprene and Ocimene-Emitting Transgenic *Arabidopsis* Plants under Contrasting Environmental Conditions

**DOI:** 10.3390/plants9040477

**Published:** 2020-04-09

**Authors:** Michele Faralli, Mingai Li, Claudio Varotto

**Affiliations:** Department of Biodiversity and Molecular Ecology, Research and Innovation Centre, Fondazione Edmund Mach, via Mach 1, 38010 San Michele all’Adige (TN), Italy; michele.faralli@fmach.it (M.F.); mingai.li@fmach.it (M.L.)

**Keywords:** isoprene, ocimene, heat stress, water stress

## Abstract

Isoprenoids are among the most abundant biogenic volatile compounds (VOCs) emitted by plants, and mediate both biotic and abiotic stress responses. Here, we provide for the first time a comparative analysis of transgenic *Arabidopsis* lines constitutively emitting isoprene and ocimene. Transgenic lines and Columbia-0 (Col-0) *Arabidopsis* were characterized under optimal, water stress, and heat stress conditions. Under optimal conditions, the projected leaf area (PLA), relative growth rate, and final dry weight were generally higher in transgenics than Col-0. These traits were associated to a larger photosynthetic capacity and CO_2_ assimilation rate at saturating light. Isoprene and ocimene emitters displayed a moderately higher stress tolerance than Col-0, showing higher PLA and gas-exchange traits throughout the experiments. Contrasting behaviors were recorded for the two overexpressors under water stress, with isoprene emitters showing earlier stomatal closure (conservative behavior) than ocimene emitters (non-conservative behavior), which might suggest different induced strategies for water conservation and stress adaptation. Our work indicates that (i) isoprene and ocimene emitters resulted in enhanced PLA and biomass under optimal and control conditions and that (ii) a moderate stress tolerance is induced when isoprene and ocimene are constitutively emitted in *Arabidopsis*, thus providing evidence of their role as a potential preferable trait for crop improvement.

## 1. Introduction

A large number of plants constitutively emit volatile organic compounds (VOCs) and it has been shown that 36% of the total photosynthetic assimilates produced by terrestrial plants are destined for VOCs’ biosynthesis [1]. The involvement of specific VOCs in a wide range of physiological processes has been largely reported [2]. Plant defense against insects, pollinator attraction, plant–plant communication, plant–pathogen interactions, reactive oxygen species scavenging, thermo-tolerance, and environmental stress adaptation are some of the most relevant ecological functions of VOCs [2]. 

Isoprene (2-methyl-1,3-butadiene, C_5_H_8_) is the most abundant naturally emitted biogenic VOC [3]. In plants, isoprene biosynthesis is catalyzed in chloroplasts by isoprene synthase (IspS) from dimethylallyl diphosphate anion (DMADP), which is formed by the 2-C-methyl-D-erythritol 4-phosphate (MEP) pathway [4]. Isoprene emission rates depend on the activity of IspS and the pool size of DMADP [5,6], which are in turn influenced by many factors, such as the endogenous developmental stage of a leaf [7,8] and several environmental stimuli and constraints [9,10]. Phylogenetic analyses show that the isoprene biosynthesis capacity was lost in *Glycine max* probably during the domestication process [11] while it was present in ancestral lines, including *Glycine soja*. Monson et al. [12] reported that isoprene emission is likely ancestral within the family Fabaceae, but several independent evolutionary events led to at least 16 losses and 10- gains in the isoprene biosynthesis capacity. The elevated frequency in gaining and losing the trait has been explained by the relatively few mutations necessary to produce or lose the IspS gene coupled with the evidence that isoprene emission is advantageous in a narrow range of environments. Recent phylogenetic reconstruction indicates that *Arundo donax* IspS (AdoIspS) and dicots IspS most likely originated by parallel evolution from Terpene Synthase b (TPS-b) monoterpene synthases, suggesting potentially different physiological roles of the two VOCs (isoprene and ocimene) under environmental stresses [13]. Therefore, understanding how isoprene affects plant growth and physiology and comparing the induced protection under abiotic stresses of isoprene and other monoterpenes will allow determination of whether isoprene emission is a beneficial trait to be reintroduced to plants, especially for the purpose of crop improvement. 

Indeed, although the MEP pathway is ubiquitous in plants, only a small portion of plants emit isoprene due to the lack of the *IspS* gene [14]. Since the biosynthesis of isoprene is a cost in terms of carbon [15,16], the great investment of energy into isoprene of some species must have relevant functional reasons. In particular, isoprene and other monoterpenes are believed to play a protective role against thermal and oxidative stresses, possibly because of the capacity of this molecule to stabilize thylakoid membranes [17,18], or to remove reactive oxygen within the mesophyll [19,20]. However, there is evidence that more stable monoterpenoids replace isoprene emission, allowing plant adaptation to more xeric environments, while isoprene emission is maintained in fast-growing plants potentially adapted to a high water availability and subjected to short and moderate stress conditions [21]. For instance, alien species of Hawaii emit more monoterpenes than native ones, and this has been suggested to be an indication of greater evolutionary success of alien species since monoterpene emission is associated with higher stress resistance [22]. More specifically, it was shown that isoprene biosynthesis evolved as an ancestral mechanism in plants to cope with transient oxidative stresses during their water-to-land transition [23]. Indeed, fast-growing hygrophilous *Quercus* species, such as most North American and some European oaks (e.g., *Quercus robur*), emit isoprene, whereas isoprene is replaced by monoterpenes in xeric oaks, such as *Q. ilex* [23,24]. In particular, it has been shown that ocimene is a commonly emitted monoterpene under stress conditions, in particular under heat stress [24]. For instance, leaves of *Quercus ilex* emit high levels of ocimene and the emission is temperature dependent and maximal at 35°C [24]. This suggests that (1) environmental conditions seem to shape isoprenoids’ emission capacity and (2) isoprene and ocimene-emitting plants may display different potential responses in stress tolerance.

To compare the role of isoprene and ocimene on environmental stress tolerance, we used some transgenic *Arabidopsis* produced in [13]. Wild-type plants and two transgenic lines per type of emitted VOC were compared in three independent experiments for their growth and stress tolerance by using a series of non-invasive shoot phenotyping techniques. This work provided for the first time a comparative analysis of *Arabidopsis* plants constitutively emitting isoprene and monoterpene regarding their growth under optimal and stress conditions and showed potential contrasting physiological behavior under disadvantageous environments.

## 2. Materials and Methods

### 2.1. Plant Materials and Growth Conditions

*Arabidopsis thaliana* L. ecotype Columbia-0 (Col-0) was used for all experiments as a wild-type control while AdoIspS-44 and AdoIspS-79 lines emitting high levels of isoprene (~300 parts per billion volume) and AdoIspS_m1-8 and AdoIspS_m1-73 lines (F310A mutation: Phenylalanine at position 310 replaced with alanine) emitting high levels of ocimene and small amounts of isoprene, were selected from a previous work [13]. All the transgenic lines overexpress the transgene under the constitutive 35S promoter in the *Arabidopsis* Col-0 background and emit the respective VOCs constitutively. Seeds of wild-type and all other lines were previously harvested from plants grown on pots in a mixture of soil (48%, 48%, and 4% of Flora gard special mixture, Einheits Erde Classic, and perlite, respectively), 23 °C temperature, 75 μmol m^−2^ s^−1^ photosynthetically active radiation (PAR), and 16/8-h light/dark photoperiod. For phenotypic and physiological characterization, seeds were germinated either on agar plates under sterile conditions (Experiment 1) or in pots (Experiment 2 and 3) in similar growing conditions to the plants used for seed collection. After seeding, agar plates or pots were stratified for 3 days in the dark at 4 °C and grown under long day conditions at 23 °C, light intensity of 75 μmol m^−2^ s^−1^ photosynthetically active radiation (PAR), and 50% relative humidity. After the full cotyledons’ emergence, seedlings were transplanted in pots containing soil and used for physiological characterization. Details on the growth conditions for each specific experiment are provided in the respective method section below.

### 2.2. Experimental Design and Stress Application

Three independent experiments were carried out in growth chambers (KBV400, BINDER GmbH, Tuttlingen, Germany). Experiment 1 was a factorial 5 × 2 experiment in a randomized block design with lines (Col-0, AdoIspS-44 and AdoIspS-79 isoprene emitters, AdoIspS_m1-8 and AdoIspS_m1-73 ocimene emitters) and watering regime (well-watered, WW and water stressed, WS) as factors in 10 blocks (*n* = 10). The experiment was set up in two identical growth chambers (KBV400, BINDER GmbH, Tuttlingen, Germany) and pots were placed in trays, with each tray containing 10 pots and treated as a block. Experiment 2 design was equal to Experiment 1, but it consisted of 12 blocks (*n* = 12) and after stress application, plants were subjected to a 7-day recovery period (fully re-watering to WS plants) and subsequent biomass harvesting. In both experiments, germinated seedlings at the two-cotyledon stage were transplanted to plastic 8x8x8 cm pots with a very similar amount of soil (~130 g of a 48%-48%-4% of Flora gard special mixture, Einheits Erde Classic, and perlite, respectively), with two per pot. Plants were subsequently thinned as one per pot according to uniform growth before the stress application. After transplanting, pots were transferred to growth chambers set at 23/22 °C daytime/nighttime temperature, an average 60% relative humidity (i.e., optimal vapor pressure deficit of ~1.1 kPa), and ~80 μmol m^−2^ s^−1^ PAR on average at the rosette level. The photoperiod was 12/12 h day/night in Experiment 1 and 10/14 h day/night in Experiment 2. The shorter photoperiod in Experiment 2 compared to Experiment 1 allowed a higher stress intensity before the onset of reproductive stages (i.e., flowering) owing to the longer vegetative phase and, therefore, total pot water loss. Pots were watered every two days to saturation to avoid soil moisture deficit. Twenty-three days after sowing, the selected pots were subjected to WS by withholding watering in both experiments. The available water content of the pots was expressed as a fraction of the transpirable soil water as FTSW= (P_g_ – P_d_)/TTSW, where (i) the total transpirable soil water (TTSW) was the difference between the pot weights at a 100% water holding capacity (WHC) (pot weight ∼230 g including the plant and plastic pot) and when the transpiration rate of the stressed plants decreased to 10% of the control plants (∼90 g), (ii) P_g_ was the actual pot weight on a given date, and (iii) P_d_ was the pot weight at the time when the transpiration rate of the stressed plants was 10% of the control plants (∼90 g of pot weight). Pot weight was assessed every day with a balance (Pioneer PA2102C, Ohaus, Parsippany, NJ, USA). 

Experiment 3 had a factorial 5 × 2 design in a randomized block with lines (as above) and temperature (control temperature (CT) and heat stress (HS)) as factors in 12 blocks (*n* = 12). For this experiment, the blocks were split in two chambers, one at the CT temperature and one at the HS temperature. Plants were transferred in pots and grown as in Experiment 2. Stress was applied 22 days after sowing and by increasing the temperature to 29/28 °C day/nighttime (standard temperature for the heat stress experiments in *Arabidopsis*, e.g., [25]) in the selected chamber devoted to HS. During the HS application, pots were watered daily to avoid a soil moisture deficit.

### 2.3. Gravimetric Assessment of Daily Transpiration

In Experiment 1 and 2 (i.e., when WS was applied), pots (*n* = 12 or 10) were weighed daily in the morning and within a 30-min time frame and from the date after treatment application (DAT) 1 on. In Experiment 3, *n* = 3 pots were used to pot FTSW daily and re-watering was carried out to avoid a soil moisture deficit. In Experiment 1 and 2, WW pots were re-watered daily to a target weight reflecting approximately 0.8–0.9 FTSW while no water was added to WS pots. The pot weights P_1_ and P_2_ of two consecutive days were used to calculate the water use of the plant over 24 h. Since soil evaporation was not minimized, empty pots were placed randomly in the growth chamber and at different FTSW to estimate the average daily evaporation, which was subtracted from the total plant water use and calculated daily plant transpiration (TR, mL day^−1^). 

### 2.4. Imaging Projected Leaf Area

For all the experiments, the projected leaf area (PLA, cm^2^) was taken for all the pots starting from DAT-1 (*n* = 10 or *n* = 12) and every two days after gravimetric assessment. Images were collected with a Samsung Galaxy A20 camera and analyzed with Easy Leaf Area software as described in [26]. Briefly, the selected pot was quickly taken from the growth chamber and placed on a table next to a red calibration area of 4 cm^2^ fixed at the top of an 8x8x8 pot (i.e., the distance between the camera and the plant/reference was identical). The picture was taken from the top at a distance of 40 cm and the camera was always positioned parallel to the plant. Image segmentation and PLA measurement was immediately carried out with the Easy Leaf Area free app and the value recorded. The PLAs P_1_ and P_2_ of two consecutive days were used to calculate the relative shoot growth rate (RGR) (% d^−1^) according to the equation: RGR = 100 × 1/*t* × ln (P_2_/P_1_), where *t* is the days between P_2_ and P_1_ (i.e., two days). 

### 2.5. Leaf number, Leaf Emergence Rate, and Phenology

In Experiment 2 and 3, the dynamic of the leaf number was characterized after shoot imaging by visually counting visible leaves (*n* = 12). Subsequently, the leaf emergence rate was calculated as the maximum slope of the linear relationship between the leaf number (LN) and time (*t*) during the experimental period (i.e., during the linear phase of plant growth). Plants were also visually inspected for phenological stages according to [27] and the date of inflorescence emergence (GS 5.10) and first flower opening (GS 6.0) were recorded. 

### 2.6. Gas-Exchange Measurements

Gas-exchange measurements (*n* = 4 to 5) were performed for Experiment 1, 2, and 3 with a Li-Cor 6400 (Li-Cor, Lincoln, NE, USA) using an integrated fluorescence leaf cuvette (LI-6400-40; Li-Cor) between 0900 and 1400. To minimize the potential leaf position and developmental stage effects, all the gas-exchange measurements were taken on the sixth fully expanded leaf of four to five randomly selected plants for each treatment. When needed, the leaf area was recalculated by imaging the portion used for gas exchange. In the Li-Cor cuvette, all the parameters (leaf CO_2_ assimilation at saturating light, *A*; and stomatal conductance, *g_s_*) were collected at 400 ppm CO_2_. Leaf temperature was maintained at 23 °C, a VPD between 0.9 and 1.3 kPa, and PAR was 600 μmol m^−2^ s^−1^ (saturating PAR for *Arabidopsis* previously evaluated by light curves (Figure S1)), with a 10:90 blue:red light and a flow rate of 400 μmol s^−1^. In Experiment 3, the block temperature was maintained either at 23 or 29 °C depending on the plant treatment (i.e., CT or HS plants). In Experiment 1, data were collected at DAT 15 (i.e., mild water stress); in Experiment 2, at DAT 22 (severe water stress); and in Experiment 3, at DAT 18.

### 2.7. A/Ci Analysis

During Experiment 2 and 3, control plants were used for further gas-exchange characterization in both Col-0 wild-type and transgenic lines (*n* = 4). Measurements of the response of *A* to sub-stomatal CO_2_ concentrations (*C_i_*) were performed using a Li-Cor 6400 (Li-Cor, Lincoln, NE, USA) and a 2-cm^2^ leaf cuvette with an integral blue–red light-emitting diode (LED) light source as described above. Cuvette conditions were maintained as described in the previous section for WS experiments. When steady-state conditions were achieved, the CO_2_ concentration was sequentially decreased to 300, 200, 150, 75, and 50 μmol mol^−1^ before returning to the initial concentration of 400 μmol mol^−1^. This was followed by a sequential increase to 500, 700, 900, 1100, 1300, and 1500 μmol mol^−1^. Readings were recorded when *A* had stabilized to the new conditions. The maximum velocity of Rubisco for carboxylation (*V_cmax_*) and the maximum rate of electron transport demand for Ribulose 1,5-bisphosphate (RuBP) regeneration (*J_max_*) were derived by curve fitting, as described by Sharkey et al. [16]. 

### 2.8. Final Biomass Assessment

In Experiment 2 and 3, on DAT 28 and 26, respectively, the shoot biomass was destructively assessed by harvesting the plants (*n* = 12). The fresh weight (FW, g) of the shoot was recorded immediately after harvest with a precision balance and samples were immediately placed inside an oven (BD115, BINDER GmbH, Tuttlingen, Germany) at 60 °C for four days. Shoot dry weight (DW, g) was then recorded by weighing the dried samples. 

### 2.9. Statistical Analysis

Statistical analyses were conducted using STATISTICA 13th (Dell Software) and RStudio (R Core Team, 2017). Randomization and experimental design were produced in RStudio with the *Agricolae* package. All the data were subjected to repeated measurement and subsequent two-way analysis of variance (ANOVA) for each DAT when line and stress factors were present (e.g., DW, PLA). Single-factor analysis was carried out with one-way ANOVA. Shapiro–Wilk and Levene’s tests were used to test data for normality and homogeneity of variance, respectively. Fisher’s least significant difference test was used for multiple comparisons. Estimation of TR_break_ was carried out with segmented regression by plotting TR and FTSW and estimated as the intersection between the two linear segments as in Faralli et al. [28].

## 3. Results

### 3.1. Stress Application and Phenology

**Figure 1:**
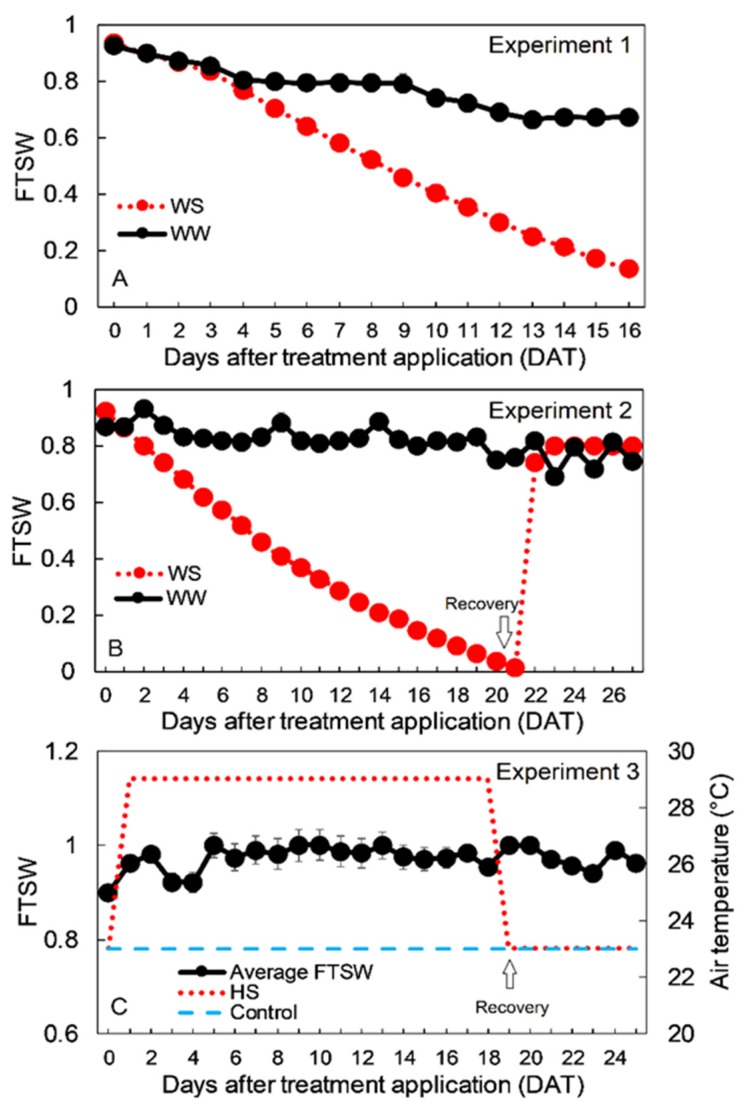
Environmental conditions for Experiment 1, 2, and 3 (**A**–**C**, respectively). In **A**, black dots represent fraction of transpirable soil water (FTSW) of well-watered (WW) plants while red dots represent FTSW for water stress (WS) plants over a 16-day experimental period (*n* = 50). In **B**, black dots represent FTSW of WW plants while red dots represent FTSW for WS plants over a 27-day experimental period (*n* = 60). From days after treatment application (DAT) 22, WS plants were subjected to a recovery period. In **C**, black dots represent the average FTSW evaluated on three average pots per treatment (*n* = 30), the red dotted line represents the average day-time air temperature of the heat stress (HS) chamber and the blue line represents the average day-time temperature of the control temperature (CT) chamber.

**Figure 2:**
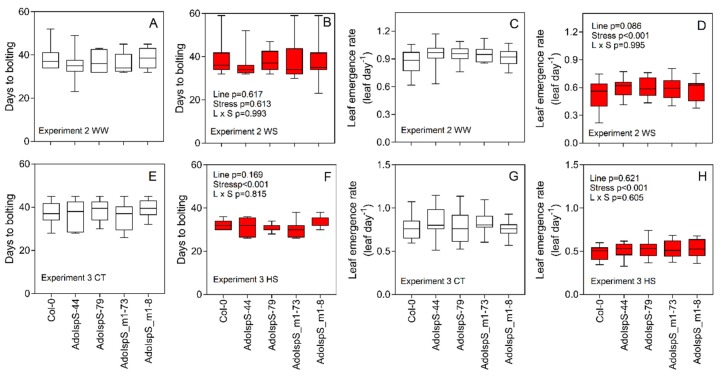
Days to bolting and the leaf emergence rate assessed in Experiment 2 (**A**–**D**) and 3 (**E**–**H**). For all the graphs, black bars represent the control (either WW or CT) conditions, whereas red bars represent plants subjected to stress (either WS or HS) (*n* = 12). Data were analyzed with two-way ANOVA and the output is included in the graph. Means separation was carried out with Fisher’s test (since no differences are present between lines, the respective letters were omitted for simplicity).

In this work, we aimed to characterize the transgenic lines under semi-realistic environmental conditions and several stressors. In Experiment 1, WS was slowly (slope linear fitting −0.053) applied for 16 days until FTSW was 0.14 on average, therefore mimicking a relatively mild WS environment (Figure 1A). Conversely, in Experiment 2, a relatively more severe stress was applied (FTSW below 0.1 for four days) followed by a re-watering period to saturation (FTSW 0.8) (Figure 1B). In Experiment 3, HS was applied by increasing the chamber temperature to 29 °C while maintaining saturating levels of the soil moisture to avoid confounding factors from WS (Figure 1C). Phenological assessments suggest that WS did not trigger an escape strategy in Experiment 2, at least at the stress conditions applied (*p* = 0.613), with similar days to bolting between WW and WS plants. Similarly, no significant differences were detected between lines in both WW and WS conditions (*p* = 0.617) (Figure 2A,B). The leaf emergence rate was around 0.85 leaf day^−1^ on average under WW conditions and this was not significantly different between lines (*p* = 0.086). Under WS, the leaf emergence rate was significantly (*p* < 0.001) lower than WW conditions for all the lines (Figure 2C,D). HS application significantly reduced the days for bolting compared with CT conditions (*p* < 0.001) (Figure 2E,F) and decreased the leaf emergence rate (*p* < 0.001) (Figure 2G,H), while no significant differences were found between lines (*p* = 0.169 and *p* = 0.621).

### 3.2. Shoot Biomass Assessment

**Figure 3:**
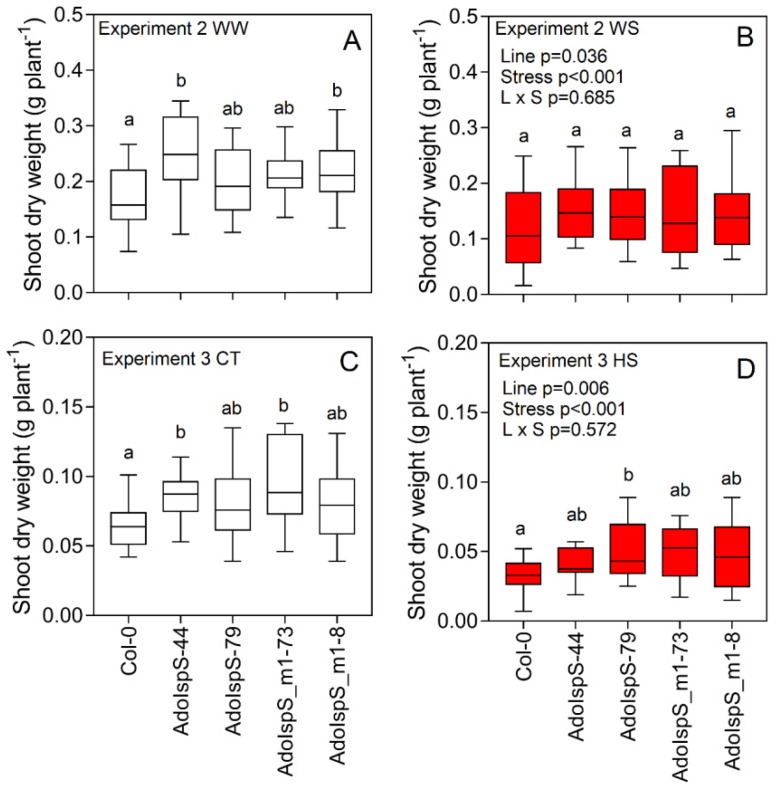
Shoot dry weight for Col-0, isoprene emitters (AdoIspS-44 and AdoIspS-79), and ocimene emitters (AdoIspS_m1-73 and AdoIspS_m1-8) *Arabidopsis* lines grown under control and stress conditions. In Experiment 2 (**A**,**B**), plants were grown under control (**A**) and water stress conditions (**B**) while in Experiment 3 (**C**,**D**), plants were grown under control (**C**, 23 °C temperature) and heat stress conditions (**D**, 29 °C temperature) (*n* = 12). The two-way ANOVA output is shown in the graph. Different letters represent significant differences according to Fisher’s test.

Under WW conditions and in Experiment 2 (50 days after seeding), Col-0 showed lower shoot dry weight biomass than AdoIspS-44 and AdoIspS_m1-8 (*p* = 0.036) (Figure 3A). WS conditions significantly (*p* < 0.001) reduced the shoot dry weight compared with the WW plants on average and for all the lines. No statistically significant differences were found between lines under WS conditions (Figure 3B). In Experiment 3 (45 days after seeding), CT Col-0 showed a reduced shoot dry weight compared to AdoIspS-44 and AdoIspS_m1-73 (*p* = 0.006) (Figure 3C). HS significantly (*p* < 0.001) reduced the shoot dry weight biomass for all the lines and by ~45% on average. When compared with Col-0 under HS, the transgenic lines showed a significantly higher dry weight biomass (*p* = 0.006) (Figure 3D)

### 3.3. Water-Use Strategies under Reduced Water Availability

**Figure 4:**
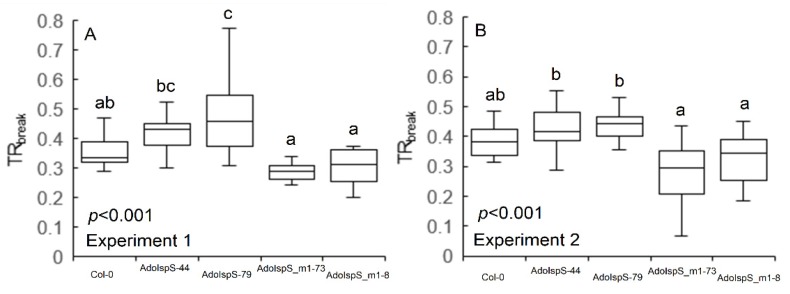
Breakpoint of plant transpiration to reduced soil water availability (TR_break_). Data were gravimetrically collected in Experiment 1 (**A**) and 2 (**B**) and daily plant transpiration was plotted against FTSW curves and subjected to segmented regression (*n* = 10 in A and *n* = 12 in B). Data were analyzed with one-way ANOVA and different letters represent significant differences according to Fisher’s test.

Under WS conditions, the reduction in transpiration for Col-0 started at FTSW of 0.35 and 0.38 for Experiment 1 and 2, respectively (Figure 4A,B). When compared with Col-0, isoprene-emitting lines (i.e., AdoIspS-44 and 79) displayed a more pronounced water conservation strategy, with a TR_break_ ranging between 0.43 and 0.47 FTSW in both experiments (*p* < 0.001). Conversely, ocimene-emitting plants showed reduced transpiration at lower FTSW compared with both Col-0 and AdoIspS lines, with a TR_break_ between 0.28 and 0.31 in both experiments (*p* < 0.001). 

### 3.4. Projected Leaf Area and Relative Growth Rate

**Figure 5:**
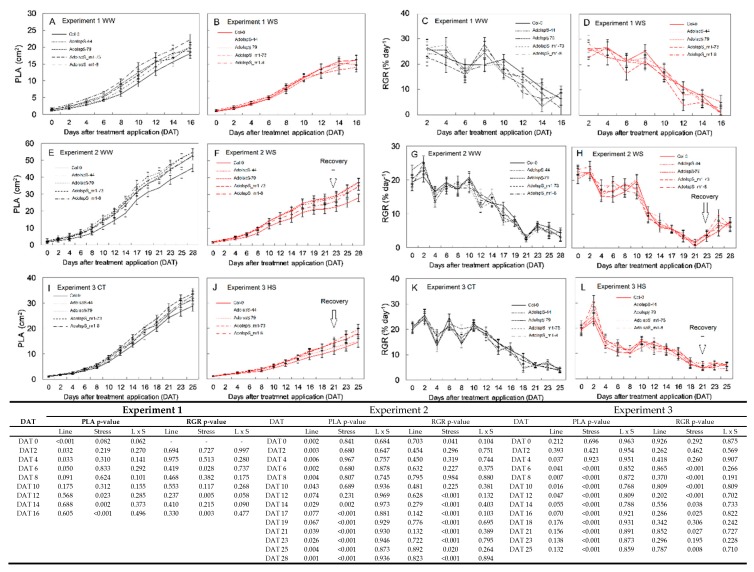
Projected leaf area (PLA) and relative growth rate (RGR) of Col-0 and isoprene- or ocimene-emitting lines. Data were collected over three experiments and during the entire experimental treatment. In (**A**,**E**,**I**), continuous assessment of PLA for control plants (WW in Experiment 1 and 2 or CT in Experiment 3) and in (**C**,**G**,**K**), the calculated RGR is shown. In (**B**,**F**,**J**), continuous assessment of PLA for stressed plants (WS in Experiment 1 and 2 or HS in Experiment 3) and in (**D**,**H**,**L**), the calculated RGR is shown. Values are means ± standard error of the means (*n* = 12 while *n* = 10 in Experiment 1). Data were subjected to repeated measurements analysis (*p* < 0.001) and two-way ANOVA and the output is shown in the table for each experiment. A multiple comparisons test (Fisher’s test) was carried out for each day after treatment (DAT) and is shown in Supplementary 2 for simplicity.

Correlations between PLA and shoot dry weight (*p* < 0.001, R^2^ = 0.84, data not shown) confirmed the reliability of the dynamic estimation of the leaf area over the experimental periods. For all the experiments under WW or CT conditions, all the transgenic lines showed larger PLA compared with the wild-type Col-0 (Figure 5A,E,I). The higher PLA relative to Col-0 was statistically significant by up to 30% for AdoIspS_m1-73 in Experiment 1 (DAT 2-6), by up to 20%–25% for AdoIspS-44 in Experiment 2 (DAT 21-28), and by up to 20% in Experiment 3 (DAT 6-12). The limitation of water availability (i.e., Experiment 1 and 2 Figure 5B,F) caused a pronounced growth retardation for all the lines (from DAT 14 both experiments), as shown by evident reductions in PLA and RGR. However, while under moderate water stress (Experiment 1), no significant differences were observed among the lines, and significantly higher PLA values for the emitters were found in Experiment 2 (severe water stress) when compared with Col-0 and during the recovery period. In particular, AdoIspS-44 and AdoIspS_m1-8 showed significantly higher PLA than Col-0 in Experiment 2, with a much sharper recovery period compared with the wild type. HS severely affected plants’ growth, with a significant reduction in PLA and RGR since DAT 6 compared with WW conditions for the lines tested. Significantly lower PLA values for Col-0 were recorded compared with emitters (e.g., AdoIspS_m1-8 DAT 25, AdoIspS_m1-73 and AdoIspS-79 DAT 21, AdoIspS_m1-73, and AdoIspS-44 DAT 8).

### 3.5. Gas-Exchange, A/Ci Analysis 

**Figure 6:**
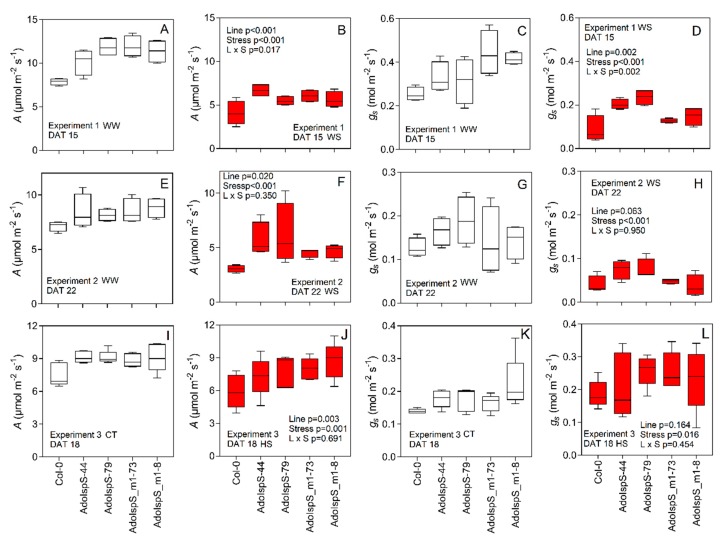
CO_2_ assimilation rate (*A*) and stomatal conductance (*g_s_*) collected in Experiment 1 (**A**–**D**), Experiment 2 (**E**–**H**), and Experiment 3 (**I**–**L**). Values are means (*n* = 4 to 5) and the two-way ANOVA output is shown in each graph. Measurements (*n* = 4 to 5) were performed at 600 μmol m^−2^ s^−1^ PAR, 23 °C leaf temperature, and 400 μmol mol^−1^ [CO_2_] in Experiment 1 and 2 (water stress experiments) while in Experiment 3 (heat stress experiment), the block temperature was 23 and 29 °C for CT and HS, respectively.

Saturating *A* for Col-0 was ~8 µmol m^−2^ s^−1^ on average, which was significantly (*p* < 0.001 and *p* = 0.003, respectively, Figure 6A,E,I) lower than transgenic lines in Experiment 1 and 3 under optimal conditions (WW and CT). Indeed, the *A*/*C_i_* analysis supports the in situ gas-exchange measurements, with both isoprene- and ocimene-emitting lines displaying higher *A* (*p* = 0.045) and *J_max_* (*p* = 0.041) while no significant differences were found for *V_cmax_* (Table 1). As expected, WS severely reduced *A* and *g_s_* and the reduction was very similar for all the lines (*p* < 0.001) (Figure 6B,F). However, in some lines (e.g., AdoIspS-44 exp 1 and ISPs 79 exp 2), higher *A* values compared with Col-0 were present. Under HS, *g_s_* was not negatively affected for all the lines, and in some cases (e.g., AdoIspS-79 and AdoIspS_m1-73), higher *g_s_* values were obtained compared with the CT (Figure 6N). On the contrary, HS reduced *A* by 20% in Col-0 (*p* = 0.016) while no significant differences compared with the WW control were recorded for ocimene emitters and AdoIspS-79 (Figure 6L).

## 4. Discussion

Transgenic approaches have been largely used to engineer isoprene emission in non-emitter species [13,19,25,29,30]. This has led to a large amount of information regarding the role of isoprene in plant growth, stress tolerance, and signaling. As of today, however, much less effort has been devoted to the comparative dissection of the differences between the biological functions of isoprene and monoterpenes. In this work, *Arabidopsis* plants transformed to emit constitutively isoprene or ocimene were compared for the first time and a comprehensive shoot characterization was carried out in order to assess their potential role on stress tolerance and plant growth. The comparative approach used in our study has two major advantages with respect to similar studies carried out in the past in different species [31]. First, it compared in the same genetic background the physiological effects of isoprene and ocimene emission, thus normalizing the effect of the starting pool of metabolites, which is known to vary among species and affect emissions [32,33]. Second, it employed two enzymes differing by only one amino acid [13], thus minimizing among transgenic emitters any confounding effects on plant growth due to the protein length or translational efficiency. Leveraging on similar emission levels from selected transgenic lines, in this study, we thus characterized the physiological effects of isoprene and ocimene under optimal conditions as well as two main abiotic stresses, temperature excess and water limitation. 

### 4.1. Hemi- and Mono-Terpene Emission Improves Plant Growth under Optimal Conditions

Under optimal conditions, isoprenoids’ emission is a metabolically expensive trait, with high energy and photosynthetic carbon requirements [15]. However, independent studies demonstrated that the emission of isoprenoids led to an increased biomass, leaf area, and pigment content in several species (e.g., [25,34]), which is consistent with our data, suggesting the existence of a tight but complex relationship between isoprene/monoterpene emission and growth. In Zuo et al. [34], *Arabidopsis* plants transformed with a *Eucalyptus globulus IspS* gene had a higher leaf area, leaf number, and final dry weight than the wild-type Col-0, consistent with our data on both isoprene and ocimene emitters. Similarly, in Loivamäki et al. [25], *Arabidopsis* lines transformed with an *IspS* gene from gray poplar had higher growth rates under optimal growth conditions. The role of isoprene as a signaling molecule has recently been shown, with a significant upregulation in the expression of genes belonging to signaling networks or associated with specific growth regulators (e.g., gibberellic acid, cytokinins, and jasmonic acid) in *Arabidopsis* engineered to emit isoprene [34]. In particular, greater accumulation of gibberellic acid, potentially through an enhanced expression level of genes encoding for zinc fingers proteins (e.g., *TZF5*), has been suggested as a potential explanation of these phenotypes with an enhanced leaf area. Additionally, isoprene appeared to enhance cytokinin levels mainly through changes to the expression levels of genes associated to cytokinin signaling. In our work, isoprene-emitting lines showed a higher PLA and final dry weight than Col-0 under optimal conditions, corroborating the hypothesis that isoprene and monoterpene emission might be involved in enhancing or modulating the gene network and signaling of plant growth. 

An interesting output of our work is the enhanced photosynthetic capacity and CO_2_ assimilation per unit of leaf area (*A*) in emitters compared with Col-0 under optimal conditions. It was previously reported that isoprene and monoterpene emission might increase the chlorophyll content in leaves, and potentially enhance *A* [25,34]. This increase in *A* can also partially explain the higher PLA and biomass of the emitters compared with Col-0, suggesting a higher carbon availability that can sustain growth. Intraspecific variation within the *Arundo* tribe and some dicots for *A* and isoprene emission revealed a positive and significant correlation between isoprene emission and photosynthesis [7,35]. Morfopoulos et al. [35] proposed a mechanism by which the isoprene emission rate is directly proportional to the excess of reducing power (nicotinamide adenine dinucleotide phosphate, NADPH) generated by the linear electron flow and unused by photosynthesis. Indeed, in our experiment, *J_max_* was significantly higher in emitters than Col-0, suggesting that a fraction of the total electron flux generated by the photosystem II might be allocated to isoprenoid biosynthesis. 

### 4.2. Isoprene and Ocimene Emission Resulted in a Moderate Tolerance to Environmental Stresses

In our work, albeit at the boundaries of significance and partly inconsistent between lines transformed with the same construct, both isoprene and ocimene emission resulted in marginally higher *A* and PLA under water and heat stress than Col-0. Conversely, significant positive effects were recorded for dry weight under heat stress only. Indeed, our data are in line with most of the literature showing the efficacy of hemi- and monoterpenes at protecting the photosynthetic apparatus under high temperatures [25,30,34]. Somehow surprisingly, however, these results indicate relatively minor phenotypic variations consequent to VOCs’ emission under the stress conditions tested. Since the heat stress regime applied in this work was milder and indubitably closer to physiological conditions than in some previous reports [30], further characterization is needed to better evaluate potentially different degrees of responses under broader environmental conditions.

However, isoprene and ocimene emitters showed an opposite behavior concerning their water-use under reduced water availability. Ocimene emitters reduced their transpiration at a very low value of FTSW, suggesting a non-conservative water-use behavior. On the contrary, isoprene emitters showed a highly conservative water-use strategy, with early stomatal closure and an elevated sensitivity of transpiration to soil drying. While dryland agriculture might benefit from conservative genotypes [28,36], a non-conservative strategy is advantageous for maximizing nutrient capture and for successful colonization of dry habitats with extreme fluctuations in resource availability [37]. Under short resource fluctuations, fast nutrient and water uptake can take over resource utilization by slower neighbors, thus providing a competitive advantage in disadvantageous epiphytic habitats [37,38]. This might indicate why monoterpene-emitting species are more common in xeric habitat than isoprene-emitting species. It was already shown that hygrophilic isoprene emitters showed elevated stomatal sensitivity to soil water stress, mainly to avoid tissue dehydration [39,40], which is consistent with our work. We speculate that the conservative behavior of the isoprene emitters analyzed in this study might also suggest a strategy to increase internal the isoprene concentration (owing to its high volatility) under stress conditions to enhance its potential beneficial effect, which is minimized under low concentrations [41]. The two contrasting strategies therefore, although they did not produce a higher dry weight biomass than Col-0 under water stress, led, for different reasons, to similar phenotypes between the two constitutively emitting lines. Further investigation on this is required in order to understand the physiological basis of this behavior and exploit the potential advantages of these responses under different magnitudes of water stress.

### 4.3. Agricultural and Evolutionary Relevance

The role of isoprenoids in plant defense strategies against biotic and abiotic stresses and their potential applications to agriculture are increasingly being appreciated [42]. Our results highlight both similarities and differences in abiotic stress tolerance among isoprene and ocimene emitters, which on the one hand determined their evolution in natural environments and on the other hand will affect the possibility of applying them to agricultural settings. Agro-ecosystems, in fact, represent simplified environments in which human beings buffer environmental conditions to provide steady and sufficient amounts of water, light, and nutrients to crops [43]. The consistently higher PLA and *A* we observed for the transgenic lines compared with Col-0 under optimal conditions recorded in this work suggest that enhanced growth is a resulting phenotype in isoprenoid-emitting plants (supported by other literature, e.g., [34]) and might be a preferable trait, at least in biomass crops. In general, irrespective of the VOC emitted, heat tolerance was generally enhanced compared with Col-0. This is relevant considering that in the future, climate change will increase the frequency of extreme weather events [44], and further suggests that enhanced isoprenoids’ emission could be a viable strategy to be used in crop improvement. For instance, under stressful conditions, the induced stress tolerance (e.g., reactive oxygen species scavenging, membrane stability, gas exchange and dry weight maintenance) and the synergistic effects between isoprenoids, secondary metabolites (e.g., carotenoids), and hormones (i.e., cytokinins) [42] is of major interest, in particular to assess whether a potential delayed in senescence is induced in isoprenoid-emitting plants under thermal stress (a favorable trait in several crops, e.g., cereals [45]). However, which terpene should be better suited to this task is still a matter of debate, as the evidence in favor of either of them is still fragmentary and partly conflicting. The presence of isoprene emission in wild soybean (*Glycine soja*) and its lack in cultivated soybean (*Glycine max*) suggests that isoprene emission in *Fabaceae* could have been counter-selected during the domestication process in favor of monoterpene emission [46]. Given the naturally occurring multiple losses and gains of isoprene emission during the course of evolution in *Fabaceae* [12], however, it is still doubtful whether IspS pseudogenization in cultivated soybean is simply a by-product or the result of domestication analogously to the loss of resistance toward pathogens observed in several other crops [47,48]. On the other hand, our results indicate that isoprene emission could be preferable over ocimene emission in the long run under the current climate change scenario, as it provides conservative water-use behavior and thus potentially higher sustainability over time [49]. From a broader evolutionary perspective, the increased sensitivity to dehydration of isoprene-emitting plants compared with ocimene emitters provides a rationale for the observation that isoprene is usually associated to a perennial lifestyle, where dehydration avoidance rather than drought escape is advantageous [50]. These results are in line with previous work suggesting that while isoprene evolved in plants adapted to high water availability and subjected to short stresses, it was replaced by monoterpenes or more stable isoprenoids in xeric environments [21]. 

## 5. Conclusions

To our knowledge, this is the first study where a comprehensive characterization of *Arabidopsis* lines constitutively emitting isoprene and ocimene has been carried out. Our data support the most recent literature on hemi- and monoterpene plant biosynthesis suggesting a positive effect of the emission on both growth and stress tolerance and corroborating the idea of their potential usefulness in crop improvement. Given the differences found in water-use strategies followed by contrasting stomatal sensitivity to water limitation, the potential application of isoprene emission for perennial crops and of monoterpene emission for annual crops will need to be assessed further. In particular, the dissection of the possible differences in the signaling cascade in isoprene and monoterpene emitters by transcriptomic approaches holds the promise to improve our understanding of the role of VOCs as signaling molecules for stress priming in plants. 

## Figures and Tables

**Table 1:** Photosynthetic to sub-stomatal CO_2_ concentration response curve (*A*/*C_i_*) output for the wild-type Col-0 and transgenic lines. Data were collected over Experiments 2 and 3 on control plants with a Licor 6400XT. Parameter estimation was carried out as described by [16]. Values are means ± standard error of the means (*n* = 4 to 5) and analyzed with one-way ANOVA while means separation was carried out by Fisher’s test.

Line	*A*	*V_cmax_*	*J_max_*
Col-0	8.0 ^a^	45.2	94.4 ^a^
AdoIspS-79	9.4 ^b^	49.1	102.3 ^a,b^
AdoIspS-44	9.6 ^b^	56.6	119.6 ^b^
AdoIspS_m1-8	9.8 ^b^	55.9	114.5 ^b^
AdoIspS_m1-73	10.2 ^b^	53.5	114.4 ^b^
		*p*-value	
	0.045	0.321	0.041

## Supplementary Materials

The following are available online at https://www.mdpi.com/2223-7747/9/4/477/s1, Figure S1: Curve of *A* to photosynthetic active radiation (*n* = 4) carried out in Col-0. Photosynthesis was fully saturated at 600 µmol m^−2^ s^−1^ PAR, Table S1: Details of statistical tests related to PLA and RGR.

## References

[1] Kesselmeier J., Ciccioli P., Kühn U., Stefani P., Biesenthal T., Rottenberger S., Wolf A., Vitullo M., Valentini R., Nobre A. (2002). Volatile organic compound emissions in relation to plant carbon fixation and the terrestrial carbon budget. Glob. Biogeochem. Cycles.

[2] Spinelli F., Cellini A., Marchetti L., Mudigere K., Piovene C. (2011). Emission and function of volatile organic compounds in response to abiotic stress. Abiotic Stress in Plants—Mechanisms and Adaptations.

[3] Guenther A., Karl T., Harley P., Wiedinmyer C., Palmer P.I., Geron C. (2006). Estimates of global terrestrial isoprene emissions using MEGAN (Model of Emissions of Gases and Aerosols from Nature). Atmos. Chem. Phys. Discuss..

[4] Schwender J., Zeidler J., Gröner R., Müller C., Focke M., Braun S., Lichtenthaler F.W., Lichtenthaler H.K. (1997). Incorporation of 1-deoxy-D-xylulose into isoprene and phytol by higher plants and algae. FEBS Lett..

[5] Wiberley A.E., Donohue A.R., Westphal M.M., Sharkey T.D. (2009). Regulation of isoprene emission from poplar leaves throughout a day. Plant Cell Environ..

[6] Rasulov B., Hüve K., Bichele I., Laisk A., Niinemets Ü. (2010). Temperature response of isoprene emission in vivo reflects a combined effect of substrate limitations and isoprene synthase activity: A kinetic analysis1. Plant Physiol..

[7] Ahrar M., Doneva D., Koleva D., Romano A., Rodeghiero M., Tsonev T., Biasioli F., Stefanova M., Peeva V., Wohlfahrt G. (2015). Isoprene emission in the monocot Arundineae tribe in relation to functional and structural organization of the photosynthetic apparatus. Environ. Exp. Bot..

[8] Niinemets Ü., Sun Z., Talts E. (2015). Controls of the quantum yield and saturation light of isoprene emission in different-aged aspen leaves. Plant Cell Environ..

[9] Loreto F., Schnitzler J.-P., Schnitzler J.-P. (2010). Abiotic stresses and induced BVOCs. Trends Plant Sci..

[10] Sharkey T.D., Monson R.K. (2017). Isoprene research—60 years later, the biology is still enigmatic. Plant Cell Environ..

[11] Sharkey T.D., Gray D.W., Pell H.K., Breneman S.R., Topper L. (2012). Isoprene synthase genes form a monophyletic clade of acyclic terpene synthases in the tps-b terpene synthase family. Evolution.

[12] Monson R.K., Jones R.T., Rosenstiel T.N., Schnitzler J.-P., Schnitzler J.-P. (2012). Why only some plants emit isoprene. Plant Cell Environ..

[13] Li M., Xu J., Alarcon A.A., Carlin S., Barbaro E., Cappellin L., Velikova V., Vrhovsek U., Loreto F., Varotto C. (2017). In planta recapitulation of isoprene synthase evolution from ocimene synthases. Mol. Boil. Evol..

[14] Loreto F., Fineschi S. (2014). Reconciling functions and evolution of isoprene emission in higher plants. New Phytol..

[15] Sharkey T.D., Wiberley A.E., Donohue A.R. (2007). Isoprene emission from plants: Why and how. Ann. Bot..

[16] Sharkey T.D., Bernacchi C., Farquhar G.D., Singsaas E. (2007). Fitting photosynthetic carbon dioxide response curves for C3leaves. Plant Cell Environ..

[17] Singsaas L., Lerdau M., Winter K., Sharkey T.D. (1997). lsoprene lncreases thermotolerance of isoprene-emitting species. Plant Physiol..

[18] Velikova V., Várkonyi Z., Szabo M., Maslenkova L., Nogues I., Kovács L., Peeva V., Busheva M., Garab G., Sharkey T.D. (2011). Increased thermostability of thylakoid membranes in isoprene-emitting leaves probed with three biophysical techniques. Plant Physiol..

[19] Vickers C.E., Possell M., Cojocariu C.I., Velikova V., Laothawornkitkul J., Ryan A., Mullineaux P.M., Hewitt C.N. (2009). Isoprene synthesis protects transgenic tobacco plants from oxidative stress. Plant Cell Environ..

[20] Loreto F., Velikova V. (2001). Isoprene produced by leaves protects the photosynthetic apparatus against ozone damage, quenches ozone products, and reduces lipid peroxidation of cellular membranes. Plant Physiol..

[21] Loreto F., Dicke M., Schnitzler J.-P., Turlings T.C.J. (2014). Plant volatiles and the environment. Plant Cell Environ..

[22] Llusià J., Peñuelas J., Sardans J., Owen S.M., Niinemets Ü. (2010). Measurement of volatile terpene emissions in 70 dominant vascular plant species in Hawaii: Aliens emit more than natives. Glob. Ecol. Biogeogr..

[23] Fini A., Brunetti C., Loreto F., Centritto M., Ferrini F., Tattini M. (2017). Isoprene responses and functions in plants challenged by environmental pressures associated to climate change. Front. Plant Sci..

[24] Loreto F., Förster A., Dürr M., Csiky O., Seufert G. (1998). On the monoterpene emission under heat stress and on the increased thermotolerance of leaves of Quercus ilex L. fumigated with selected monoterpenes. Plant Cell Environ..

[25] Loivamäki M., Gilmer F., Fischbach R.J., Sörgel C., Bachl A., Walter A., Schnitzler J.-P. (2007). Arabidopsis, a model to study biological functions of isoprene emission?. Plant Physiol..

[26] Bloom A.J. (2014). Easy leaf area: Automated digital image analysis for rapid and accurate measurement of leaf area. Appl. Plant Sci..

[27] Boyes U.C., Zayed A.M., Ascenzi R., McCaskill A.J., Hoffman N.E., Davis K., Görlach J. (2001). Growth stage –based phenotypic analysis of arabidopsis. Plant Cell.

[28] Faralli M., Williams K.S., Han J., Corke F.M.K., Doonan J.H., Kettlewell P.S. (2019). Water-saving traits can protect wheat grain number under progressive soil drying at the meiotic stage: A phenotyping approach. J. Plant Growth Regul..

[29] Sharkey T.D., Yeh S., Wiberley A.E., Falbel T.G., Gong D., Fernandez D.E. (2005). Evolution of the isoprene biosynthetic pathway in Kudzu1[w]. Plant Physiol..

[30] Sasaki K., Saito T., Lämsä M., Oksman-Caldentey K.-M., Suzuki M., Ohyama K., Muranaka T., Ohara K., Yazaki K. (2007). Plants utilize isoprene emission as a thermotolerance mechanism. Plant Cell Physiol..

[31] Feng Z., Yuan X., Fares S., Loreto F., Li P., Hoshika Y., Paoletti E. (2019). Isoprene is more affected by climate drivers than monoterpenes: A meta-analytic review on plant isoprenoid emissions. Plant Cell Environ..

[32] Nogues I., Brilli F., Loreto F. (2006). Dimethylallyl diphosphate and geranyl diphosphate pools of plant species characterized by different isoprenoid emissions1. Plant Physiol..

[33] Owen S.M., Peñuelas J. (2013). Volatile isoprenoid emission potentials are correlated with essential isoprenoid concentrations in five plant species. Acta Physiol. Plant..

[34] Zuo Z., Weraduwage S.M., Lantz A.T., Sanchez L.M., Weise S.E., Wang J., Childs K.L., Sharkey T.D. (2019). Isoprene acts as a signaling molecule in gene networks important for stress responses and plant growth. Plant Physiol..

[35] Morfopoulos C., Prentice I.C., Keenan T.F., Friedlingstein P., Medlyn B.E., Peñuelas J., Possell M. (2013). A unifying conceptual model for the environmental responses of isoprene emissions from plants. Ann. Bot..

[36] Sinclair T.R., Hammer G.L., Van Oosterom E.J. (2005). Potential yield and water-use efficiency benefits in sorghum from limited maximum transpiration rate. Funct. Plant Boil..

[37] Querejeta J.I., Prieto I., Torres M.P., Campoy M., Alguacil M.D.M., Roldan A. (2018). Water-spender strategy is linked to higher leaf nutrient concentrations across plant species colonizing a dry and nutrient-poor epiphytic habitat. Environ. Exp. Bot..

[38] Chesson P., Gebauer R.L.E., Schwinning S., Huntly N., Wiegand K., Ernest S.K.M., Sher A., Novoplansky A., Weltzin J.F. (2004). Resource pulses, species interactions, and diversity maintenance in arid and semi-arid environments. Oecologia.

[39] Tattini M., Loreto F., Fini A., Guidi L., Brunetti C., Velikova V., Gori A., Ferrini F. (2015). Isoprenoids and phenylpropanoids are part of the antioxidant defense orchestrated daily by drought-stressed Platanus × acerifolia plants during Mediterranean summers. New Phytol..

[40] Velikova V., Brunetti C., Tattini M., Doneva D., Ahrar M., Tsonev T., Stefanova M., Ganeva T., Gori A., Ferini F. (2016). Physiological significance of isoprenoids and phenylpropanoids in drought response of Arundinoideae species with contrasting habitats and metabolism. Plant Cell Environ..

[41] Harvey C., Li Z., Tjellström H., Blanchard G.J., Sharkey T.D. (2015). Concentration of isoprene in artificial and thylakoid membranes. J. Bioenerg. Biomembr..

[42] Brilli F., Loreto F., Baccelli I. (2019). Exploiting Plant Volatile Organic Compounds (VOCs) in agriculture to improve sustainable defense strategies and productivity of crops. Front. Plant Sci..

[43] Robertson G.P., Swinton S.M. (2005). Reconciling agricultural productivity and environmental integrity: A grand challenge for agriculture. Front. Ecol. Environ..

[44] Porter J.R., Semenov M. (2005). Crop responses to climatic variation. Philos. Trans. R. Soc. B Boil. Sci..

[45] Cossani C.M., Reynolds M. (2012). Physiological traits for improving heat tolerance in wheat. Plant Physiol..

[46] Lantz A.T., Allman J., Weraduwage S.M., Sharkey T.D. (2019). Isoprene: New insights into the control of emission and mediation of stress tolerance by gene expression. Plant Cell Environ..

[47] Rosenthal J.P., Dirzo R. (1997). Effects of life history, domestication and agronomic selection on plant defence against insects: Evidence from maizes and wild relatives. Evol. Ecol..

[48] Rodriguez-Saona C., Vorsa N., Singh A.P., Johnson-Cicalese J., Szendrei Z., Mescher M.C., Frost C.J. (2011). Tracing the history of plant traits under domestication in cranberries: Potential consequences on anti-herbivore defences. J. Exp. Bot..

[49] Nakhforoosh A., Bodewein T., Fiorani F., Bodner G. (2016). Identification of water use strategies at early growth stages in durum wheat from shoot phenotyping and physiological measurements. Front. Plant Sci..

[50] Turner N.C. (1986). Adaptation to water deficits: A changing perspective. Funct. Plant Boil..

