# Supplementary material for: Shoot Characterization of Isoprene and Ocimene-Emitting Transgenic Arabidopsis Plants under Contrasting Environmental Conditions
Source: Plants (Basel). 2020 Apr 9;9(4):477. doi: 10.3390/plants9040477 (PMC7238224; doi:10.3390/plants9040477)
Supplement: Supplementary file 1 [file plants-09-00477-s001.zip › Supplementary_1.docx]

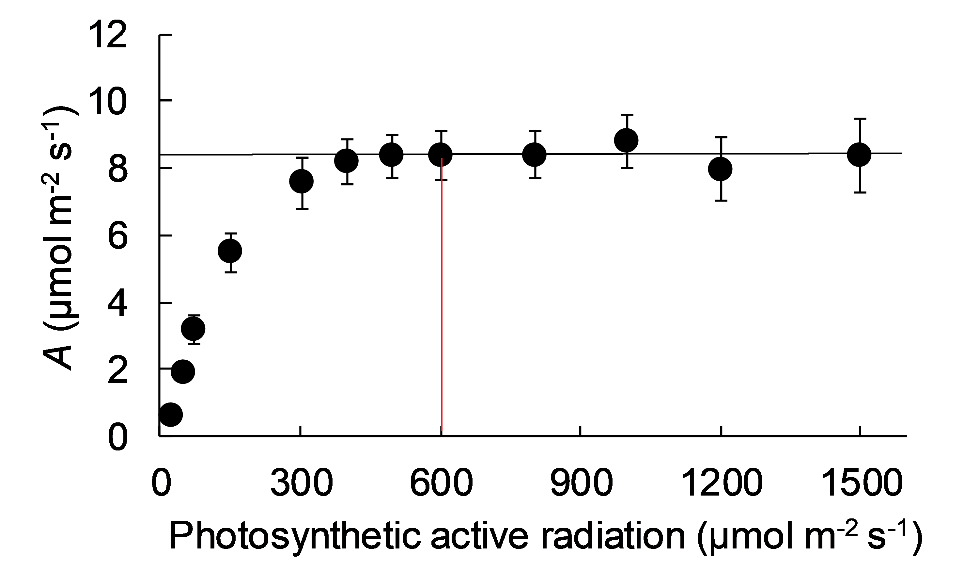


Supplementary figure 1. Curve of *A* to photosynthetic active radiation (n=4) carried out in Col-0. Photosynthesis was fully saturated at 600 micromole PAR.
